# Non-Isothermal Crystallization Kinetics of Montmorillonite/Polyamide 610 Nanocomposites

**DOI:** 10.3390/nano13121814

**Published:** 2023-06-06

**Authors:** Yang Fu, Cuimeng Huo, Shuangyan Liu, Keqing Li, Yuezhong Meng

**Affiliations:** 1 High & New Technology Research Center of Henan Academy of Sciences, Zhengzhou 450002, China; fuyang@hnas.ac.cn (Y.F.); liushuangyan@hnas.ac.cn (S.L.); likeqing@hnas.ac.cn (K.L.); 2 Institute of Chemistry Co., Ltd., Henan Academy of Sciences, Zhengzhou 450002, China; huocuimeng@hnas.ac.cn; 3 Research Center of Green Catalysts, College of Chemistry, Zhengzhou University, Zhengzhou 450001, China; 4 Institute of Chemistry, Henan Academy of Sciences, Zhengzhou 450000, China; 5 State Key Laboratory of Optoelectronic Materials and Technologies, Sun Yat-Sen University, Guangzhou 510275, China

**Keywords:** non-isothermal crystallization, montmorillonite, polyamide 610, polymeric nanocomposites

## Abstract

Non-isothermal crystallization kinetics of montmorillonite (MMT)/polyamide 610 (PA610) composites were readily prepared by in situ melt polymerization followed by a full investigation in terms of their microstructure, performance, and crystallization kinetics. The kinetic models of Jeziorny, Ozawa, and Mo were used in turn to fit the experimental data, in all of which Mo’s analytical method was found to be the best model for the kinetic data. Differential scanning calorimetry (DSC) and transmission electron microscopy (TEM) studies were used to investigate the isothermal crystallization behavior and MMT dispersion levels in the MMT/PA610 composites. The experiment results revealed that low MMT content can promote the PA610 crystallization, whilst high MMT content result in MMT agglomeration, and reduce the PA610 crystallization rate.

## 1. Introduction

Polyamide, as an engineering plastic, is an important member of the polymer family with several commercial applications in recent decades. Polyamides are semi-crystalline polymers that have good mechanical strength, great abrasion resistance, and a high modulus. Thus, they are commonly employed in fibers and thermoplastics because of this. The physicochemical qualities and strength of semi-crystalline polymers are well known to be highly dependent on their crystalline shape, structure, and degree of crystallization [1,2,3]. The investigation of polyamide crystallization kinetics about material parameters aids in controlling the crystallization rate and crystallinity of polyamides and obtaining the required qualities [4,5,6,7].

Polyamide 610 (PA610, H-[HN(CH_2_)_6_NHCO(CH_2_)_8_CO]_n_-OH) is widely used for automobile manufacturing [8], electronic [9,10,11], and machinery industries [12,13,14] applications, and for the production of a wide range of biological materials [15,16]. The main chain backbone of PA610 contains multiple carbonyl and secondary amino groups forming numerous intramolecular and intermolecular hydrogen bonds [17] that contribute to its crystalline structure [18,19].

The crystallization kinetics of PA610–have an important effect on its morphology and mechanical properties. In addition, extrusion, injection molding, film-forming, and fiber-forming techniques that are used to process polyamides normally occur via non-isothermal processes [20,21]. Therefore, it is necessary to study the non-isothermal crystallization kinetics of PA610 to establish a theoretical background to optimize PA610 polymer processing conditions. Studies on the crystallization performance of PA610 polymers produced in non-isothermal crystallization processes have previously been carried out on polymer composites containing relatively filler content of 0.2~0.5 wt% [22,23] and on polymer blends [24,25]. MMT influences polymer crystallization in a non-homogeneous nucleation manner, which changes polymer strength [26]. Dai et al. investigated the non-isothermal crystallization behavior of MMT/iPP composites (MMT content: 5 wt%) [27]. They demonstrated that adding MMT with strong nucleation activity might lower the crystallization activation energy of iPP while increasing its crystallization rate. By using MMT as a crystallization aid (MMT content: 1 wt%), Xue et al. investigated the crystallization behavior of PTT/PC blends [28]. They discovered that uniformly exfoliated and distributed MMT contributed considerably to the crystallization of PTT. However, there were a few reports on the crystallization performance of polyamides produced in non-isothermal crystallization processes containing relatively large amounts of filler (e.g., 1% to 10% filler content). High-content and high-span MMT/polyamide composites are more commonly employed in practice.

In practice, the composite with higher filler content is of more interest. For organic fillers, Jape et al. [29] found that low concentrations of organic filler in nylon 66/thermotropic liquid crystalline polymers (LCP) promoted crystallization, while higher LCP concentrations inhibited the crystallization. For the case of inorganic fillers, Liu [30] et al. employed melt blending to prepare Mg(OH)_2_/PA6 composites, reporting that the crystallization performance of the composites containing 60 wt% Mg(OH)_2_ system was better than those containing higher Mg(OH)_2_ content (>70 wt%). Wang [31] et al. also used melt-impregnation processes to fabricate polyamide 6 (PA6)/long carbon fiber (LCF) composites containing 50 wt% LCF, demonstrating that the LCF component performed an important role in the heterogeneous nucleation processes. 

Herein, we report the non-isothermal crystallization behavior of PA610 containing different amounts of montmorillonite (MMT) filler. The crystallization kinetics of these composite processes have been analyzed using the methods of Jeziorny, Ozawa, and Mo [32,33,34] modifications of Avrami’s equation [35], with Mo’s method providing the most accurate description of the non-isothermal crystallization process of the MMT/PA610 composites.

## 2. Materials and Methods

### 2.1. Instrumentation and Reagents

#### 2.1.1. Reagents

Aqueous PA610 solution (60 wt%) was purchased from Pingdingshan Shenma Engineering Plastics Co. Ltd., Pingdingshan, China. Hypophosphite was obtained from Beijing Hengye COSCO Chemical Co. Ltd., Beijing, China. Modified MMT was prepared with the reported procedure [34]. 

#### 2.1.2. Instrumentation

Thermal characterization of the MMT/PA610 composites was performed using a modulated differential scanning calorimeter (DSC, Q2000, TA Instruments, New Castle, DE, USA). Thermogravimetric analysis (TGA) was conducted on a TGA8000 instrument (Perkin-Elmer) using a heating rate of 10 °C min^−1^ under an N_2_ atmosphere. Transmission electron microscopy (TEM) analyses were conducted using a JEM-1200EX electron microscope (Jeol). A 6-L pressurized reaction kettle fitted with a safe operating system, purchased from Weihai Zhenhong Chemical Machinery Co., Ltd., Weihai, China, was used for polymerization studies. 

### 2.2. Experimental Details

#### 2.2.1. Preparation of MMT/PA610 Composites

(1) Different amounts of MMT filler were dispersed in rapidly stirred solutions of 60 wt% PA610 to provide samples PA-1, PA-5, and PA-10 containing 1, 5, and 10% of MMT, respectively.
(2) Each of the PA610 samples were transferred to a pressure reactor under an N_2_ atmosphere before adding sodium hypophosphite (0.2 wt%) as the chain transfer catalyst to initiate the polymerization. The pressure of the sealed reactor was monitored until it reached 0.2–0.3 MPa, then the reaction mixture was stirred rapidly at 85–87 rpm min^−1^.
(3) The heated 220–240 °C, and the pressure was maintained at 1.9–2.5 Mpa for 2.5–3 h.
(4) The reactor temperature was increased to 240–255 °C while the reactor pressure was gradually released to atmosphere pressure for 1–1.5 h.
(5) The temperature of the reactor was further increased to 260–270 °C for 20–30 min at atmospheric pressure.
(6) Once the temperature of the reactor reached 270–280 °C, the stirred reactor was evacuated to produce a pressure of 0.008–0.01 Mpa for 10–30 min. Stirring was stopped once the torque of the stirring paddle reached 2.5–3 Nm, and the resultant composites were obtained.

#### 2.2.2. DSC Analyses

Non-isothermal crystallization processes of the different PA610 composites were analyzed under nitrogen at a flow rate of 20.0 mL min^−1^. Samples were heated from RT to 280 °C at a heating rate of 10 °C min^−1^. The temperature was held for 10 min to eliminate the sample’s thermal histories. Samples were then cooled to RT at different cooling rates of 5, 10, 15, 20, and 25 °C min^−1^, respectively. The samples were then reheated from RT to 280 °C at a heating rate of 10 °C min^−1^.

#### 2.2.3. Calculation of Composite Crystallinity Levels

The absolute crystallinity X_c_ value of composite samples was calculated using Equation (1):

(1) $$X_{c}=\left(\frac{{\Delta H}_{m}\;}{{\Delta H}_{m}^{⊖}}\right)\times 100\%$$

where ΔH_m_ is the melting enthalpies of the composites obtained from the DSC curves and Δ$H_{m}^{⊖}$ is the standard melting enthalpy of 100% crystalline PA610 (197 J g^−1^).

The relative crystallinity (X_T_) at temperature (varies with temperature) of the composites was determined using Equation (2):

(2) $$X_{T}=\frac{\int_{T_{0}}^{T}\frac{\mathrm{dH}}{\mathrm{dT}}\mathrm{dT}}{\int_{T_{0}}^{T_{\infty }}\frac{\mathrm{dH}}{\mathrm{dT}}\mathrm{dT}}=\frac{A_{T}}{A_{\infty }}$$

where T_0_ and T_∞_ are the temperatures at the beginning and end of the crystallization process, respectively; T is the crystallization temperature; and A_T_ and A_∞_ are the crystallization peak areas for the temperature ranges of T_0_~T and T_0_~T_∞_, respectively. 

Therefore, the analysis of the crystallization exotherm curves at different cooling rates produces the relative crystallinity value, X_T_, at temperature T.

These temperatures can be converted into a time scale using Equation (3):

(3) $$t=\frac{T_{0}-T}{\Phi }$$

where T_0_ is an arbitrary reference melting temperature and Φ is the cooling rate. 

This enables the relative crystallinity as a function of time to be calculated. Temperatures were taken from the time-point when the relative crystallinity value was ~50%. By combining this temperature value with Equation (3), the half crystallization time (t_1/2_) value was calculated.

## 3. Results and Discussion

### 3.1. Morphological and Compositional Characterization of the MMT/PA610 Nanocomposites

**Figure 1:**
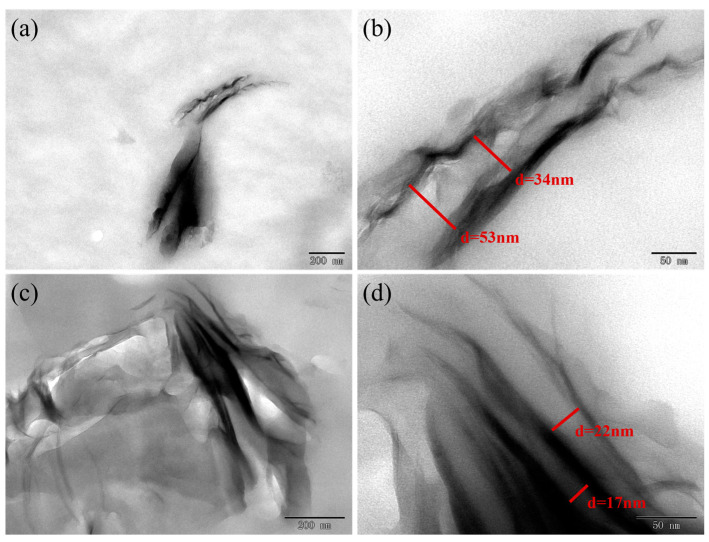
TEMs of MMT/PA610 composites: (**a**) TEM image of composite containing 1 wt% MMT; (**b**) Enlargement of TEM image shown in 1(**a**); (**c**) TEM image of composite containing 10 wt% MMT; (**d**) Enlargement of TEM image shown in 1(**c**).

The dispersity of the filler in the composite had an important effect on the crystalline properties. Figure 1 shows the TEM images of MMT/PA610 composites containing MMT of 1 and 10 wt%, respectively. MMT was found to be well dispersed throughout the composite in the 1 wt% MMT composite, which gave relatively large layer spacing (Figure 1a,b). However, the MMT component in the 10 wt% composite was agglomerated and formed a relatively small interlayer spacing (Figure 1c,d). We believe that well-dispersed MMT facilitates the crystallization of PA610, while agglomerated MMT has a hindering effect on the crystallization of PA610. This will be demonstrated later in the model analysis.

**Figure 2:**
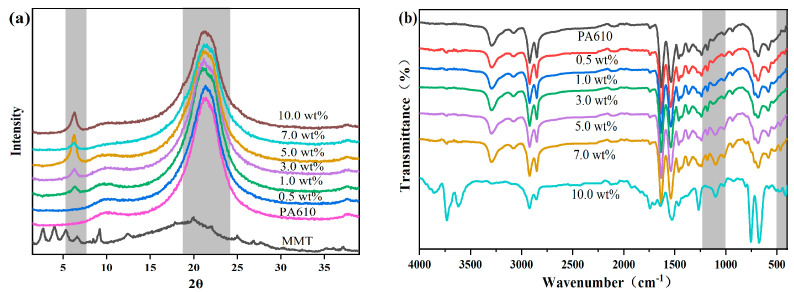
(**a**) XRD and (**b**) FT−IR spectra of pure PA610 and MMT/PA610 composite with w(MMT) = 0.5%, w(MMT) = 1.0%, w(MMT) = 3.0%, w(MMT) = 5.0%, w(MMT) = 7.0%, w(MMT) = 10.0%.

Figure 2a shows the XRD spectra of different MMT/PA610 composites. The diffraction peaks at 2θ = 21.6° correspond to the (100) crystal plane of nylon, indicating that the addition of MMT does not alter the triclinic crystal system of α-PA610. The difference in peak height and width proves the crystallinity was changed by the addition of MMT. In addition, the composites obtained with w(MMT) greater than or equal to 1.0% observed a characteristic diffraction peak of MMT at 2θ = 6.3°, indicating PA610 is intercalated effectively in MMT layers. When w(MMT) increased to 5.0% or even higher, MMT would aggregate, and the intercalation of PA in MMT was reduced.

Fourier transform infrared spectra (FTIR) of pure PA610 and different MMT/PA610 composites are presented in Figure 2b. The characteristic peaks at 1541, 1630, and 1633 cm^−1^ correspond to the N−H deformation vibration, the N−H stretching vibrations, and C−O stretching vibrations of the amide (−CONH−) groups, respectively, while the peak at 1373 cm^−1^ originates from N−H bending vibrations and C−N stretching vibrations of −CONH− groups. The characteristic bands at 2852 and 2923 cm^−1^ refer to the stretching vibration peaks of methylene (−CH_2_−) and methyl (CH_3_−) groups, respectively. 

With the addition of MMT, the wide band between 455 and 477 cm^−1^ becomes visible, which is attributed to the characteristic absorption peak of the Al−O bond. Meanwhile, the characteristic absorption peak of the Si−O bond was observed at 1027 cm^−1^ to further verify the presence of MMT. As the amount of MMT increases, the Si−O absorption peak increases, indicating the successful formation of MMT/PA610 composites.

### 3.2. Crystallization Behavior of MMT/PA610 Composite Produced at Different Cooling Rates

**Figure 3:**
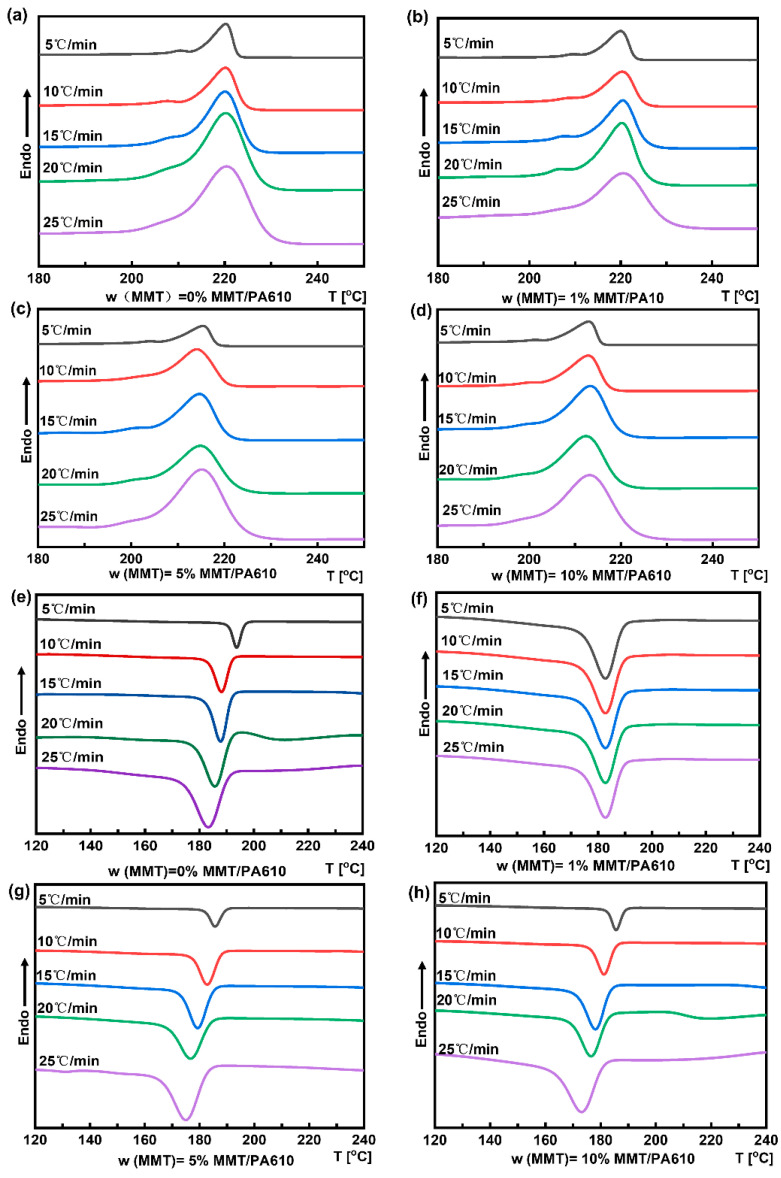
DSC curves of MMT/PA610 composite produced at different cooling rates: (**a**) Cooling curves for pure PA610; (**b**) Cooling curves of the composite containing 1 wt% MMT; (**c**) Cooling curves of the composite containing 5 wt% MMT; (**d**) Cooling curves of the composite containing 10 wt% MMT; (**e**) Heating curves of pure PA610; (**f**) Heating curves of the composite containing 1 wt% MMT; (**g**) Heating curves of the composite containing 5 wt% MMT; and (**h**) Heating curves of the composite containing 10 wt% MMT.

Composites may exhibit different crystallization behaviors with different cooling rates during the crystallization process. Figure 3 shows the DSC curves of PA610 and the MMT/PA610 composites, whose non-isothermal crystallization parameters are reported in Table 1. For pure PA610, both T_0_ and T_∞_ values were found to increase as the cooling rate increased. This is consistent with the fast crystallization due to the relatively uniform crystal nucleation with more rapid cooling resulting in faster crystal nucleation over a relatively narrow crystallization peak range. However, for MMT/PA610 composites, T_0_ and T_∞_, values were lower at faster cooling rates, with MMT promoting the crystal nucleation processes at lower crystallization temperatures. A rapid drop in temperature during the PA610 crystallization process results in a lag in the rate of incorporation of PA610 molecules into the crystal lattice, so the crystallization peak temperature was lower at a slower cooling rate. Higher MMT content resulted in the composite with a lower melting point (for the same heating rate). The DSC melting curve exhibited two peaks at a slow heating rate and showed a single peak at a faster heating rate. Two DSC peaks are likely attributed to two different crystalline forms.

Moreover, the crystallization behavior of MMT/PA610 composite with w(MMT) = 3.0% is observed by Transmission Polarizing Microscope (TPM) in Figure S1. Comparing the image which cools to (a) 30 °C with (b) −40 °C, it can be concluded that, as the temperature decreases, the crystallinity of the material increases, and the grains become smaller and finer. The addition of MMT, as confirmed, results in a decrease in its crystallinity.

### 3.3. Crystallinity Temperatures and Time Curves of the MMT/PA610 Composites

Table 1 shows that the increase in the cooling rate for pure PA610 resulted in an increase in T_0_ and T_p_ with a narrower crystallization peak range. This confirms that more rapid cooling produced a higher crystallization temperature due to the increased rate of uncontrolled crystal nucleation. Conversely, increasing the cooling rate for the MMT/PA610 composites resulted in an overall decrease in their T_0_ and T_p_ values. This suggests that the low content of MMT (e.g., 1 wt%) performed a role in facilitating crystal nucleation of PA610 as the temperature dropped, leading to slower and more controlled crystallization processes with an overall lower crystallization temperature. However, a higher level of MMT (e.g., 5 wt%) in the composites resulted in MMT agglomeration, which reduced its ability to facilitate the nucleation of PA610 with a reduced crystallization temperature.

**Figure 4:**
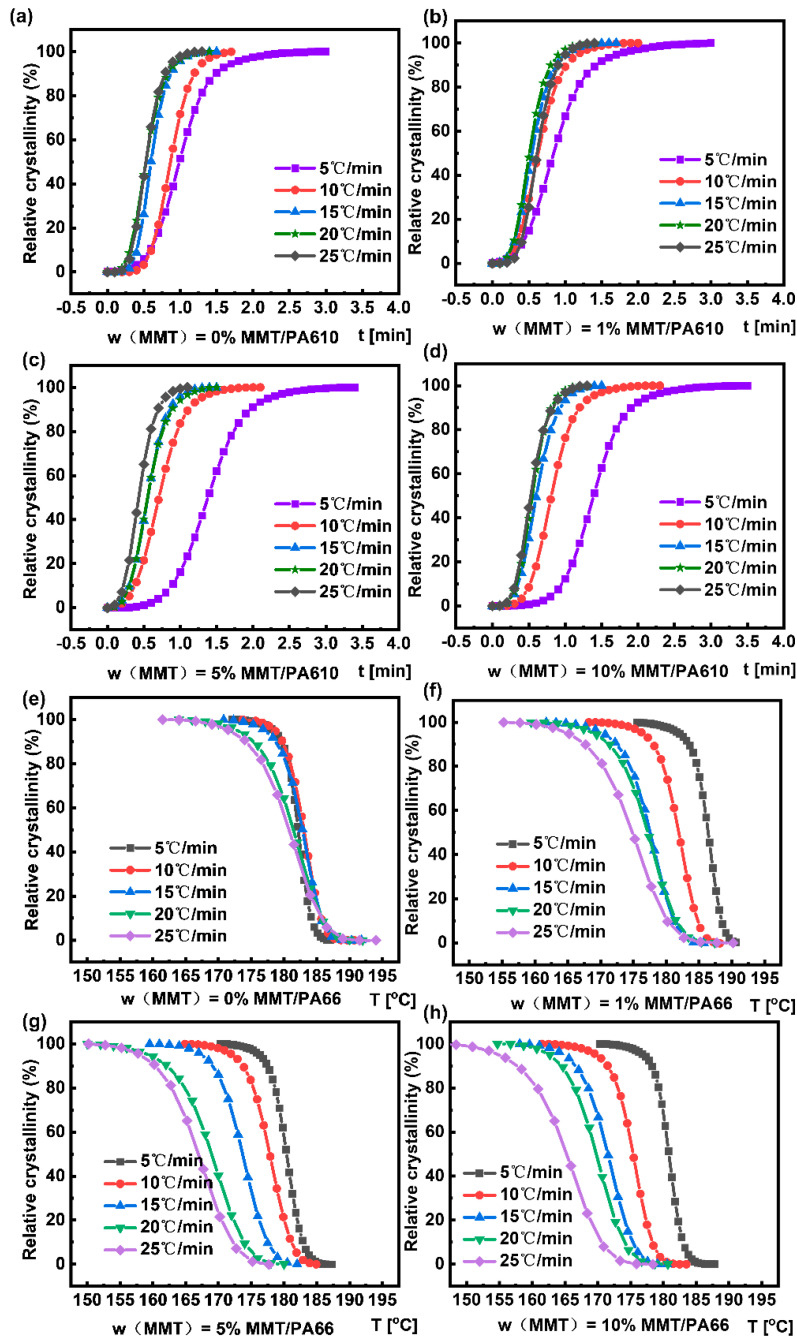
Graphs of relative crystallinities vs. temperatures for composites produced at different cooling rates: (**a**) Pure PA610; (**b**) MMT/PA610 composite containing 1 wt% MMT; (**c**) MMT/PA610 composite containing 5 wt% MMT; (**d**) MMT/PA610 composite containing 10 wt% MMT; Graphs of relative crystallinities versus times for composites produced at different cooling rates: (**e**) Pure PA610; (**f**) MMT/PA610 composite containing 1 wt% MMT; (**g**) MMT/PA610 composite containing 5 wt% MMT; (**h**) MMT/PA610 composite containing 10 wt% MMT.

Figure 4 shows the relative crystallinity as a function of temperature and time for composites at different cooling rates. From the curves, it is possible to determine the t_1/2_ at 50% relative crystallinity, which was used to calculate the crystallization rate constants *G* = (t_1/2_)^−1^. For better comparison, the TG results of MMT/PA610 composites with different content of MMT under air atmosphere are shown in Figure S2. It is obvious that the weight loss curves of the pyrolysis process are basically consistent with a significant decomposition of the main structure at around 450 °C. Table 1 reveals that, in general, a greater *G* value can be achieved with a faster crystallization heating rate, Φ. However, with low MMT content (e.g., 1 wt%), and the increase in the crystallization heating rate, the crystallization rate constant was initially increased to its maximum value at 20 °C min^−1^. A further increase in the heating rate caused the decrease in the crystallization rate constant. On the other hand, with a higher MMT content (e.g., 10 wt%), the crystallization rate constants were monotonically increased as the heating rates were increased. For pure PA610, a relatively low crystallization rate indicates that PA610 deposition cannot compete with the rapid decrease in temperature. The inclusion of MMT resulted in a more efficient nucleation process, which increased the rate of crystallization of PA610.

### 3.4. Analysis of Non-Isothermal Crystallization Processes

#### 3.4.1. Jeziorny’s Method for Analyzing MMT/PA610 Composites

Avrami proposed Equation (4), also commonly known as the Johnson–Mehl–Avrami–Kolmogorov (JMAK) theory, was used for analyzing the isothermal crystallization kinetics of composites:

(4) $$\mathrm{lg}\left[-\ln\left(1-X_{t}\right)\right]=\mathrm{lgZ}+\mathrm{nlgt}$$

Jeziorny then modified Avrami’s isothermal crystallization analysis using Equation (5) to include a correction factor for the cooling rate to enable an analysis of non-isothermal processes [32]:

(5) $${\mathrm{lgZ}}_{c}=\mathrm{lgZ}/\Phi$$

where Z_c_ is the Jeziorny crystallization rate constant after cooling rate correction and Φ is the cooling (or heating) rate.

After the inclusion of this correction, the Avrami equation can be represented as Equation (6), which enables the apparent Avrami index *n* and crystallization rate parameters Z and Z_c_ to be calculated.

(6) $$\mathrm{lg}\left[-\ln\left(1-X_{t}\right)\right]={\mathrm{lgZ}}_{c}+\mathrm{nlgt}$$

**Figure 5:**
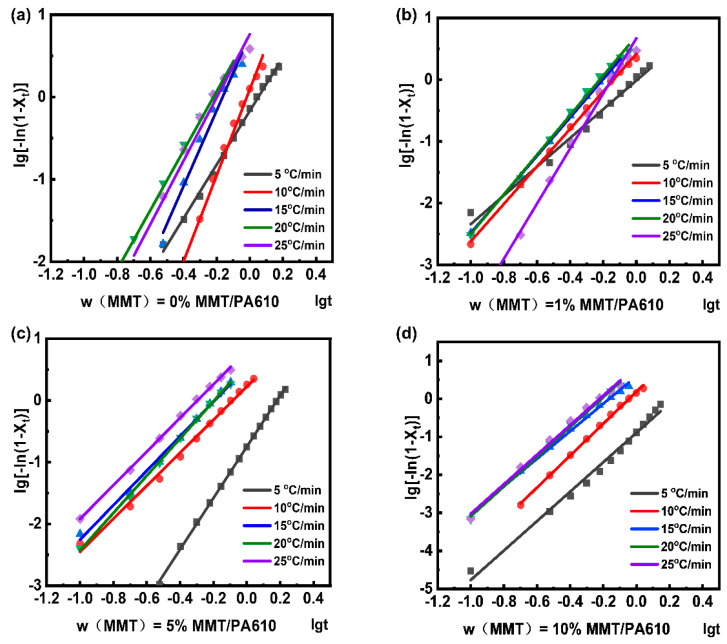
Relation curves about lg[−ln(1 − X_t_)] and lgt for: (**a**) pure PA610; (**b**) MMT/PA610 composite with w(MMT) = 1%; (**c**) MMT/PA610 composite with w(MMT) = 5%; (**d**) MMT/PA610 composite with w(MMT) = 10%.

The crystalline behaviors of the composites analyzed by the Jeziorny method are shown in Figure S3. The results fitted in the Jeziorny model are shown in Figure 5 with the parameters listed in Table 2. Since the Jeziorny method is only capable of analyzing the crystallization behavior of the composite during the initial phase of crystallization, it fails to provide information on the initial and later stages of the secondary crystallization phase.

For the same sample, the *Z_c_* values increased as cooling rates increased, suggesting a greater overall crystallization rate (Table 2). At the lowest cooling rate of 5 °C min^−1^, all samples have a Z_c_ less than 1. However, by increasing the cooling rate to 25 °C min^−1^, the Z_c_ values were increased to 5.75, 4.68, 6.46, and 7.41 for the samples of PA610, PA-1, PA-5, and PA-10, respectively. With n values between 3 and 4, the composite materials tend to exist as a mixture of two and three-dimensional crystallization.

#### 3.4.2. Ozawa’s Method for Analyzing MMT/PA610 Composites

Ozawa extended the Avrami method of composite analysis to take into account the effect of cooling rates on non-isothermal polymer nucleation and crystallization [33]. They introduced the use of a modified Equation (7) to study the rate of polymer crystallization as the function of temperature:

(7) $$\mathrm{lg}\left[-\ln\left(1-X_{T}\right)\right]=\mathrm{lgK}\left(T\right)-\mathrm{mlg}\Phi$$

where m is the Ozawa exponent that is dependent on the crystal growth and nucleation mechanism, and K(T) is the kinetic crystallization rate constant.

**Figure 6:**
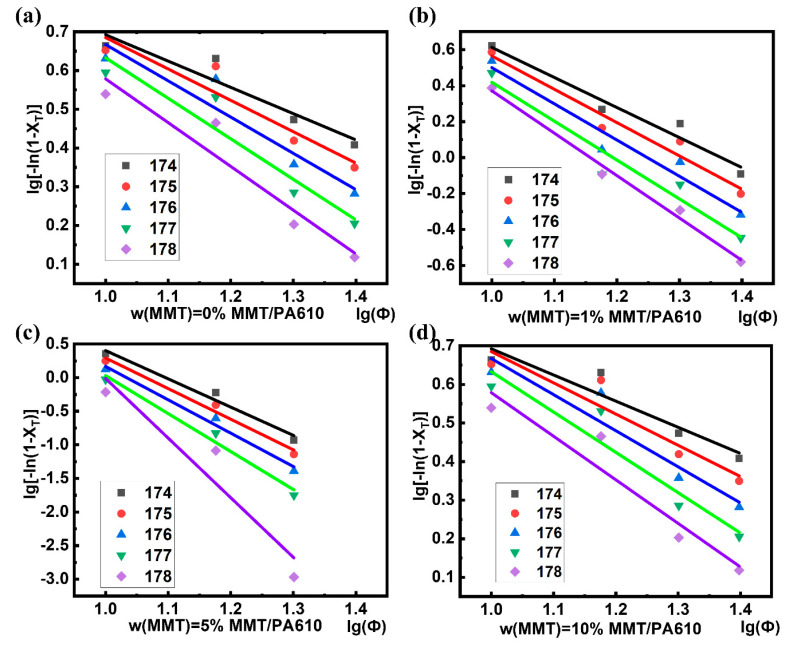
Relation curves between lg[−ln(1−X_T_)] and lgΦ for (**a**) pure PA610, (**b**) MMT/PA610 composite containing 1 wt% MMT; (**c**) MMT/PA610 composite containing 5 wt% MMT; (**d**) MMT/PA610 composite containing 10 wt% MMT.

The non-isothermal crystalline behavior of the composite materials produced in this study was analyzed using the Ozawa method, as described in Figure S4. Ozawa’s method normally produces a straight-line curve whose slope corresponds to the Ozawa coefficient m. However, from the data reported in Figure S4, the treatment of the kinetic data for the MMT/PA610 composites using Ozawa’s method produced divergent results. Further analysis of the non-isothermal crystallization data of MMT/PA610 composites for a cooling rate of 5 °C min^−1^ (Figure 6 and Table 3) revealed that curve variance, R^2^, with the Ozawa method was significant. This indicates that the Ozawa method is not suitable for studying the crystallization behavior of these types of composites due to the secondary crystallization process that occurred in MMT/PA610 polymerization process.

#### 3.4.3. Mo’s Method for Analyzing MMT/PA610 Composites

**Figure 7:**
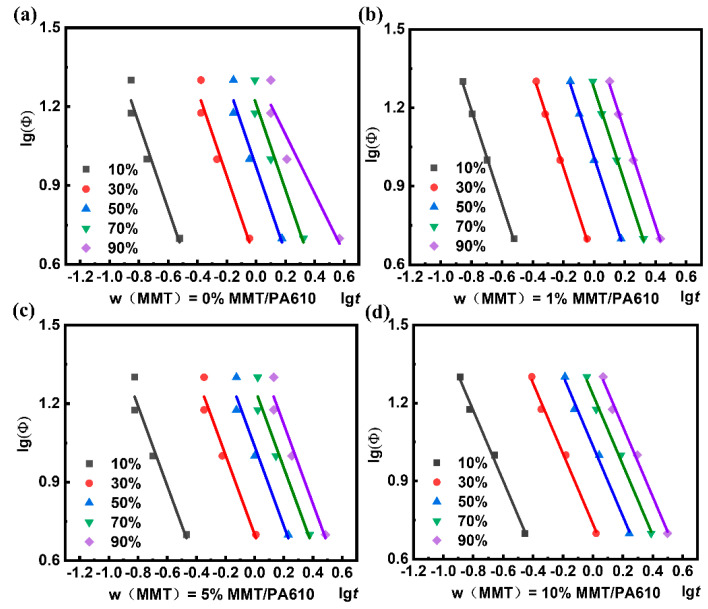
Relation curves between lgΦ and lgt for: (**a**) pure PA610, (**b**) MMT/PA610 containing 1 wt% MMT; (**c**) MMT/PA610 containing 5 wt% MMT; (**d**) MMT/PA610 containing 10 wt% MMT. (Not including cooling data of 25 °C min^−1^).

Ozawa’s method employs the relative crystallinity value of a composite at a specific temperature. This approach is not well-suited to analyzing non-isothermal crystallization processes [23]. Mo et al. [34] addressed these issues by combining the methods of Jeziorny and Ozawa to obtain Equation (8):

(8) $$\mathrm{lg}\Phi =\mathrm{lgF}\left(T\right)-\mathrm{algt}$$

where a = n/m is the dimension of crystallization, and F(T) is the cooling rate required to reach a certain relative crystallinity over a defined period of time, which exhibits a negative correlation with the crystallization rate. The non-isothermal crystallization curve analyzed using Mo’s method is shown in Figure S5. At the cooling rate of 25 °C min^−1^, a large deviation in the fitted curves was observed and so this data was removed (Figure 7 and Table 4).

F(T) in Table 4 is a measure of the crystallization rate for the composites that exhibit different relative crystallinities. A smaller F(T) value indicates a higher crystallization rate, and the increase in F(T) represents the increased levels of relative crystallinity. This means that a decreased crystallization rate offers an increase in the relative crystallinity, implying that the increased cooling rates during later stages of crystallization are associated with higher crystallinity levels. The F(T) of the composite material containing 1% MMT was lower than that of pure PA610 at an initial crystallization, which became higher at a higher level of crystallization, implying that low amounts of MMT result in decreased PA610 crystallization rates producing higher relative crystallinities. This is also reflected in the change in the absolute crystallinity (Xc) of the composite material (Table 1). The addition of a large amount of MMT was shown to hinder PA610 crystallization significantly, with the value of pure PA610 fluctuating greatly, whilst the value of the MMT/PA610 composites remained unchanged (Table 4). The agglomeration behavior of the composite containing a high MMT content has an unfavorable influence on the crystallization of PA610. The TEM images also confirmed this, which agrees with the Mo analyses. Overall, the Mo model was found to provide the best explanation of the non-isothermal crystallization data produced from the PA610/MMT composites.

## 4. Conclusions

The non-isothermal crystallization kinetics of MMT/PA610 nanocomposites prepared by in situ melt polymerization have been studied using DSC. First, we observed the dispersed behavior of MMT in the nanocomposites by TEM and found that 1 wt% MMT was well dispersed throughout the composite, but 10 wt% MMT resulted in agglomeration. Combining methods of XRD and FT-IR, we further confirmed the formation of MMT/PA610 nanocomposites and positive intercalation effect with an MMT content not exceeding 5 wt%. Then, TPM and DSC were both used to investigate the crystallization behavior of MMT/PA610 composite. It indicates that MMT can shift the crystal nucleation processes to lower crystallization temperatures, leading to a decrease in crystallinity. The results of the model analysis showed that the nanocomposite with low amounts of MMT would result in decreased PA610 crystallization rates and produce higher relative crystallinities, whilst that with larger amounts of MMT hindered crystallization of the PA610 nanocomposites. Presumably, this originated with the agglomeration behavior of MMT. Different methods were used to analyze the kinetic data produced in this study, and the Mo method could accurately describe the non-isothermal crystallization behavior of the MMT/PA610 composite. This result can provide a reference for the processing of polyamides with high content of inorganic fillers in actual production.

## Figures and Tables

**Table 1:** Non-isothermal crystallization parameters of PA610 and MMT/PA610 composites.

Samples	Φ [°C min^−1^]	T_0_ [°C]	T_p_ [°C]	T_m_ [°C]	t_1/2_ [min]	G [min^−1^]
PA610	5	190.6	183.3	214.9	1.69	0.59
	10	191.7	185.7	213.6	0.86	1.16
	15	191.8	187.7	213.4	0.60	1.68
	20	192.0	188.1	213.0	0.53	1.90
	25	196.5	193.7	212.3	0.63	1.60
PA-1	5	194.6	191.6	214.0	1.64	0.61
	10	192.2	188.1	213.7	1.03	0.97
	15	190.2	185.1	213.4	0.84	1.19
	20	189.4	183.6	213.3	0.61	1.64
	25	189.0	182.8	212.4	0.61	1.63
PA-5	5	189.3	185.7	208.7	1.77	0.56
	10	187.9	182.8	207.6	1.00	1.00
	15	185.0	179.3	206.5	0.75	1.33
	20	184.0	176.8	206.6	0.75	1.33
	25	182.7	175.1	206.4	0.63	1.59
PA-10	5	188.8	185.6	205.6	1.58	0.63
	10	185.5	181.3	204.8	1.01	0.99
	15	183.6	178.1	204.0	0.80	1.24
	20	182.7	176.6	205.2	0.64	1.56
	25	180.9	173.2	204.4	0.62	1.61

Note: t_1/2_, G, and X_1/2_ represent half crystallization time lengths, reciprocals of half crystallization times, and absolute crystallinity values, respectively. T_0_, T_p_, and T_m_ represent the initial crystallization, the crystallization peak, and the initial melting temperature, respectively.

**Table 2:** Non-isothermal crystallization kinetic parameters of MMT/PA610 composite calculated using the Jeziorny method.

Sample	Φ [°C min^−1^]	n	lgZ_c_	Z_c_	R^2^
PA610	5	3.26	−0.17	0.68	1.00
	10	5.22	0.09	1.23	0.99
	15	4.59	0.75	5.62	0.98
	20	3.61	0.78	6.03	1.00
	25	3.85	0.76	5.75	0.98
PA-1	5	2.33	−0.01	0.98	0.99
	10	3.04	0.41	2.57	1.00
	15	3.11	0.63	4.27	1.00
	20	3.20	0.70	5.01	1.00
	25	4.46	0.67	4.68	0.99
PA-5	5	4.15	−0.75	0.18	1.00
	10	2.67	0.22	1.66	0.99
	15	2.79	0.53	3.39	1.00
	20	3.02	0.59	3.89	1.00
	25	2.72	0.81	6.46	1.00
PA-10	5	3.87	−0.89	0.13	0.99
	10	4.20	0.19	1.55	1.00
	15	3.62	0.59	3.89	1.00
	20	3.92	0.83	6.76	1.00
	25	3.89	0.87	7.41	0.99

Note: Φ, n, and R^2^ represent cooling rate, Avrami index, and variance values, respectively.

**Table 3:** Non-isothermal crystallization kinetic parameters for MMT/PA610 composites calculated using the Ozawa method.

Sample	T [°C]	m	lgK(T)	R^2^
PA610	174	0.68	1.37	0.90
	175	0.81	1.49	0.90
	176	0.94	1.60	0.91
	177	1.05	1.68	0.91
	178	1.13	1.71	0.92
PA-1	174	1.67	2.28	0.96
	175	1.85	2.42	0.96
	176	2.01	2.51	0.95
	177	2.16	2.58	0.94
	178	2.36	2.73	0.99
PA-5	174	4.20	4.61	0.98
	175	4.56	4.85	0.98
	176	4.96	5.13	0.97
	177	5.65	5.68	0.98
	178	8.87	8.86	0.91
PA-10	174	0.68	1.37	0.90
	175	0.81	1.49	0.90
	176	0.94	1.60	0.90
	177	1.05	1.68	0.91
	178	1.13	1.71	0.92

Note: m corresponds to the Ozawa coefficient.

**Table 4:** Non-isothermal crystallization kinetic parameters for MMT/PA610 composites calculated using Mo’s method.

Sample	X_t_	lgF(t)	F(t)	a	R^2^
PA610	10%	−0.17	0.676083	1.63	0.95
	30%	0.61	4.073803	1.63	0.95
	50%	0.97	9.332543	1.63	0.95
	70%	1.21	16.2181	1.35	0.95
	90%	1.32	20.89296	1.12	0.92
PA-1	10%	−0.25	0.562341	1.80	1.00
	30%	0.61	4.073803	1.80	1.00
	50%	1.01	10.23293	1.80	1.00
	70%	1.27	18.62087	1.80	1.00
	90%	1.46	28.84032	1.80	1.00
PA-5	10%	−0.03	0.933254	1.52	0.95
	30%	0.69	4.897788	1.52	0.95
	50%	1.03	10.71519	1.52	0.95
	70%	1.26	18.19701	1.52	0.95
	90%	1.42	26.30268	1.52	0.95
PA-10	10%	0.09	1.230269	1.35	0.99
	30%	0.73	5.370318	1.35	0.99
	50%	1.03	10.71519	1.35	0.99
	70%	1.23	16.98244	1.35	0.99
	90%	1.37	23.44229	1.35	0.98

Note: X_t_, a, and R^2^ represent relative crystallinities at a certain time, Mo coefficients, and variance levels, respectively.

## Data Availability

Data are contained within the article.

## Supplementary Materials

The following are available online at https://www.mdpi.com/article/10.3390/nano13121814/s1. Figure S1: Transmission polarizing microscope of MMT/PA610 composite with w(MMT) = 3.0% which slowly cools to (a) 30 °C, and (b) −40 °C, respectively. Figure S2: TG curves of: (a) pure PA610, and MMT/PA610 composite with (b) w(MMT) = 1.0%, (c) w(MMT) = 3.0%, (d) w(MMT) = 5.0%, (e) w(MMT) = 7.0%, (f) w(MMT) = 10.0%. Figure S3: The Jeziorny method diagram of MMT/PA610 before optimization. Figure S4: Ozawa method diagram of MMT/PA610 composite material. Figure S5: Mo method diagram of MMT/PA610 composite.
