# Supplementary material for: Non-Isothermal Crystallization Kinetics of Montmorillonite/Polyamide 610 Nanocomposites
Source: Nanomaterials (Basel). 2023 Jun 6;13(12):1814. doi: 10.3390/nano13121814 (PMC10302243; doi:10.3390/nano13121814)
Supplement: Supplementary file 1 [file nanomaterials-13-01814-s001.zip › nanomaterials-2387196-supplementary.pdf]

SUPPORTING INFORMATION：

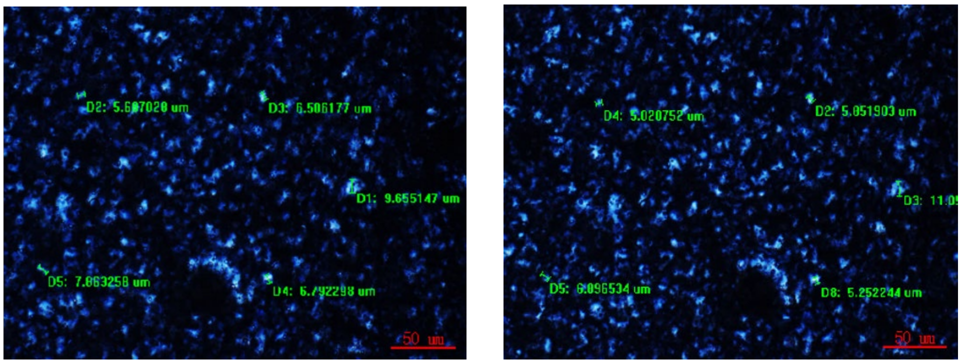

Figure S1. Transmission polarizing microscope of MMT/PA610 composite with w(MMT)=3.0% which slowly cools to (a) 30 °C, and (b) -40 °C, respectively.

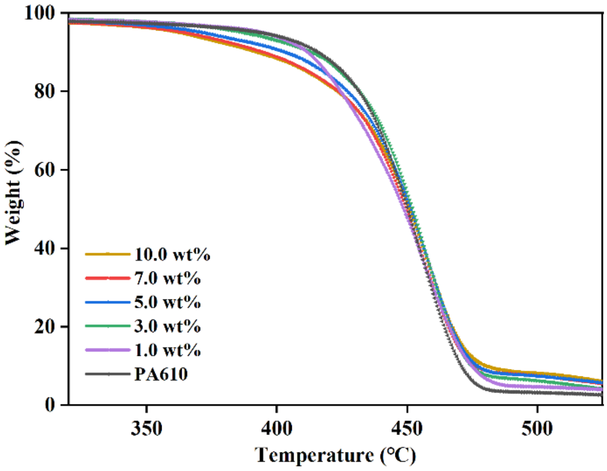

Figure S2. TG curves of: (a) pure PA610, and MMT/PA610 composite with (b) w(MMT)=1.0%, (c) w(MMT)=3.0%, (d) w(MMT)=5.0%, (e) w(MMT)=7.0%, (f) w(MMT)=10.0%

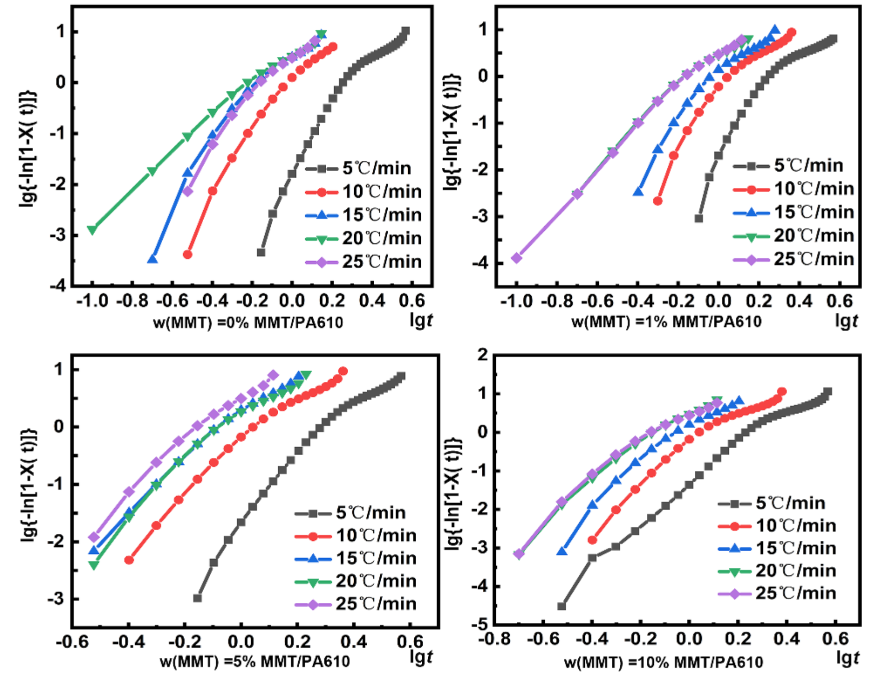

Figure S3. The Jeziorny method diagram of MMT/PA610 before optimization

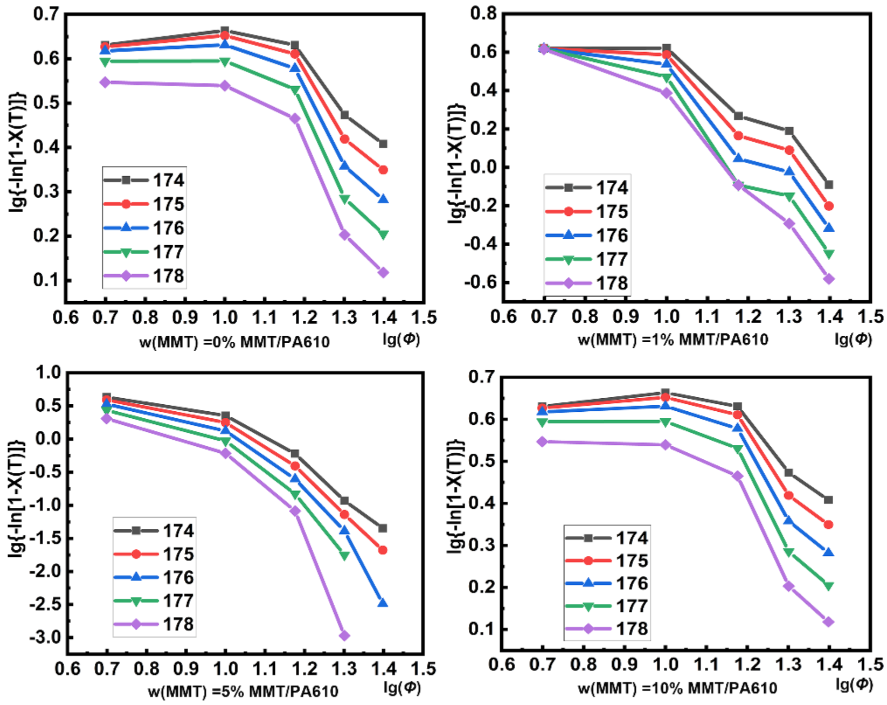

Figure S4. Ozawa method diagram of MMT/PA610 composite material

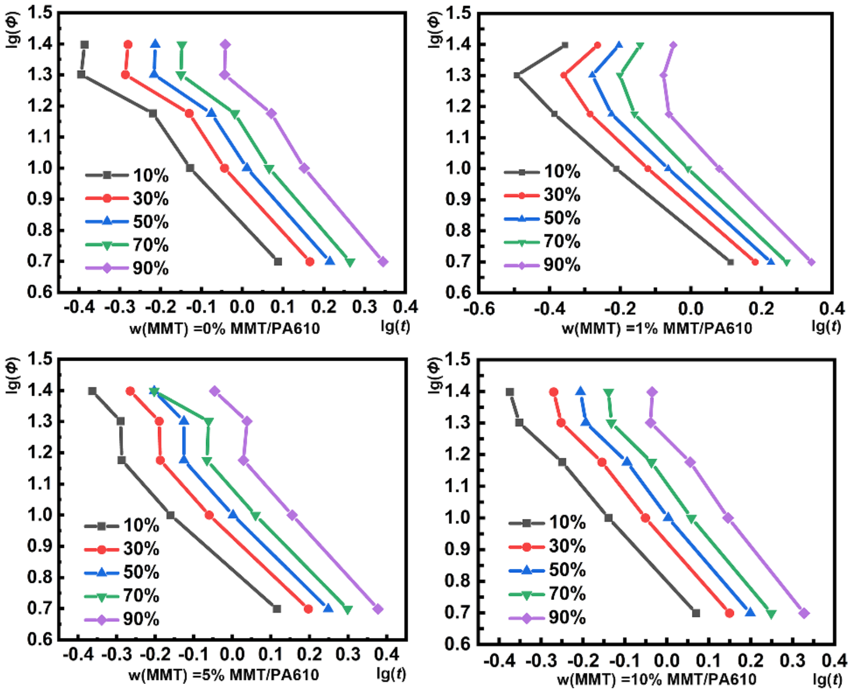

Figure S5. Mo method diagram of MMT/PA610 composite
